# Reply to Wostyn et al.: Potential models for perivascular space (PVS) enlargement and spaceflight-associated neuro-ocular syndrome (SANS)

**DOI:** 10.1073/pnas.2208241119

**Published:** 2022-07-18

**Authors:** Giuseppe Barisano, Elena Tomilovskaya, Donna R. Roberts, Floris L. Wuyts

**Affiliations:** ^a^Laboratory of NeuroImaging, University of Southern California, Los Angeles, CA 90033;; ^b^Institute of Biomedical Problems, Russian Academy of Sciences, Moscow 123007, Russia;; ^c^Department of Radiology, Medical University of South Carolina, Charleston, SC 29425;; ^d^Lab for Equilibrium Investigations and Aerospace, University of Antwerp, B-2610 Antwerp, Belgium

We thank Wostyn et al. (1) for their supportive comments, their interest in our research (2), and their informative descriptions of the potential mechanisms underlying the observed spaceflight-related changes in perivascular spaces (PVS). We also appreciate their intriguing question about the possibility to determine whether the MRI-visible PVS measured in our study corresponded to periarterial or perivenous spaces. With the current MRI data collected in astronauts and cosmonauts, it was not possible for us to reliably discriminate the periarterial from the perivenous compartment. At least another sequence for specifically imaging arteries (e.g., MR angiography) or veins (e.g., susceptibility-weighted sequences) would be required to define the detected MRI-visible PVS as periarterial or perivenous. Recent MRI studies found that the majority of MRI-visible PVS in the white matter are periarterial (3–5). The reason why perivenous spaces are less visible than periarterial spaces on structural MRI data remains currently elusive, but a few potential explanations include their smaller size and/or a different composition of the perivenous fluid compared with the periarterial. A very recent pathological study in patients with cerebral amyloid angiopathy also showed that the pathologically enlarged PVS are mostly periarterial (6).

Based on these findings, it is conceivable that the MRI-visible PVS that we quantified in astronauts and cosmonauts are mostly periarterial.

Interestingly, increased stiffness and reduced compliance in arteries above the heart have been described after long-duration spaceflight (7), resulting in reduced arterial wall compliance and pulsatility, a major motive force for the perivascular fluid (8). The accumulation of fluid in periarterial spaces might contribute to reduce the arterial pulsatility, due to the increased resistance on the arterial wall, possibly leading to further stagnation of perivascular fluid in a feedforward loop and to a reduction in perfusion, as described in a terrestrial analog for spaceflight (9).

In addition to the mechanisms underlying PVS changes, it is also important to understand the clinical consequences of these alterations to the space flyers. Increased blood biomarkers of brain injury after long-duration spaceflight have been recently discovered (10), which could derive from a potentially compromised perivascular clearance pathway, as suggested by the PVS enlargement we observed (2).

Moreover, we found an association between a higher MRI-visible PVS volume in white matter, both preflight and postflight, and the development of the spaceflight-associated neuro-ocular syndrome (SANS) (2), suggesting that some individuals with a higher baseline amount of fluid in PVS might have an increased risk for SANS. Together with lateral ventricles, PVS might represent a buffering system for the brain fluids, and the PVS buffering capacity might have a volumetric threshold above which the spaceflight-associated cerebral fluid accumulation could induce structural changes in the eyes.

Our results further highlight the importance of implementing advanced MRI sequences (11) to investigate the brain vasculature, the perivascular compartments, and the cerebrospinal fluid dynamics in space flyers for a better understanding of the brain modifications related to prolonged microgravity and their reversibility after returning on Earth, and for the development of strategies to improve and facilitate the human adaptation to space.
